# Preservation of Organ Function in Locally Advanced Non-Metastatic Gastrointestinal Stromal Tumors (GIST) of the Stomach by Neoadjuvant Imatinib Therapy

**DOI:** 10.3390/cancers13040586

**Published:** 2021-02-03

**Authors:** Nikolaos Vassos, Jens Jakob, Georg Kähler, Peter Reichardt, Alexander Marx, Antonia Dimitrakopoulou-Strauss, Nils Rathmann, Eva Wardelmann, Peter Hohenberger

**Affiliations:** 1 Mannheim University Medical Center, Division of Surgical Oncology and Thoracic Surgery, University of Heidelberg, 68167 Mannheim, Germany; nikolaos.vassos@umm.de; 2 Mannheim University Medical Center, Department of Surgery, University of Heidelberg, 68167 Mannheim, Germany; georg.kaehler@umm.de; 3 Department of General, Visceral and Pediatric Surgery, University Medical Center Göttingen, 37073 Göttingen, Germany; jens.jakob@med.uni-goettingen.de; 4 Department of Oncology and Palliative Care, Helios Klinikum Berlin-Buch, 13125 Berlin, Germany; peter.reichardt@helios-gesundheit.de; 5 Mannheim University Medical Center, Institute of Pathology, University of Heidelberg, 68167 Mannheim, Germany; alexander.marx@umm.de; 6 German Cancer Research Center (DKFZ), Clinical Cooperation Unit Nuclear Medicine, 69210 Heidelberg, Germany; a.dimitrakopoulou-strauss@dkfz.de; 7 Institute of Clinical Radiology and Nuclear Medicine, Mannheim University Medical Center Mannheim, University of Heidelberg, 68167 Mannheim, Germany; nils.rathmann@umm.de; 8 Gerhard-Domagk-Institute of Pathology, University Hospital Münster, 48149 Münster, Germany; eva.wardelmann@ukmuenster.de

**Keywords:** gastrointestinal stromal tumor, GIST, stomach, neoadjuvant therapy, imatinib, organ preservation

## Abstract

**Simple Summary:**

This study reports a single-center analysis of 55 patients with primary, locally advanced gastric GIST treated with imatinib mesylate (IM) preoperatively for a median of 10 months. The therapy yielded shrinkage of median tumor size from 113 mm to 62 mm. This facilitated 50 patients to undergo significantly less-extensive surgical procedures and resulted in a stomach preservation rate of 96%. The rate of R0 resections was 94% and was followed by a mean recurrence-free-survival time of 132 months with the median not reached. The approach was successful even for patients starting IM during an episode of upper gastrointestinal bleeding. Neoadjuvant IM therapy for locally advanced, non-metastatic gastrointestinal stromal tumors (GIST) of the stomach may play an important role in preserving organ function which might be important for IM plasma levels in an adjuvant or metastatic setting.

**Abstract:**

Background: Neoadjuvant imatinib mesylate (IM) for advanced, non-metastatic gastrointestinal stromal tumors (GIST) of stomach is recommended to downsize the tumor prompting less-extensive operations and preservation of organ function. Methods: We analyzed the clinical-histopathological profile and oncological outcome of 55 patients (median age 58.2 years; range, 30–86 years) with biopsy-proven, cM0, gastric GIST who underwent IM therapy followed by surgery with a median follow-up of 82 months. Results: Initial median tumor size was 113 mm (range, 65–330 mm) and 10 patients started with acute upper GI bleeding. After a median 10 months (range, 2–21 months) of treatment, tumor size had shrunk to 62 mm (range, 22–200 mm). According to Response Evaluation Criteria In Solid Tumors version 1.0 and version 1.1 (RECIST 1.1), 39 (75%) patients had partial response and 14 patients had stable disease, with no progressive disease. At plateau response, 50 patients underwent surgery with an R0 resection rate of 94% and pathological complete response in 24%. In 12 cases (24%), downstaging allowed laparoscopic resection. The mean recurrence-free survival (RFS) was 123 months (95%CI; 99–147) and the estimated 5-year RFS was 84%. Conclusions: Neoadjuvant IM allowed stomach preservation in 96% of our patients with excellent long-term RFS, even when starting treatment during an episode of upper GI bleeding. Preservation of the stomach provides the physiological basis for the use of oral IM in the adjuvant or metastatic setting.

## 1. Introduction

Gastrointestinal stromal tumors (GISTs) are the most common mesenchymal tumors of the gastrointestinal tract, arising mostly in the stomach [1,2]. GISTs exhibit a broad spectrum of clinical behavior [2,3,4] and are characteristically driven by activating mutations of KIT- or platelet-derived growth factor receptor-a (PDGFR-a) gene in approximately 85–90% of cases [5,6]. Surgery was the mainstay of curative treatment of GIST [7,8]. Since 2001, the natural history of GISTs has been dramatically altered through the use of imatinib mesylate (IM), a receptor tyrosine kinase inhibitor of KIT [9,10,11]. Imatinib is approved for treatment of metastatic or unresectable GISTs and for adjuvant therapy after R0 resection of GIST with significant risk of metastatic spread [11,12].

Although the majority of GISTs are resectable at presentation, a significant number of GISTs are either locally advanced, requiring challenging and complex operations which can lead to postoperative morbidity [3], or the tumors also might present as primarily not resectable with clear margins; however, debulking procedures are difficult to perform due to the high vascularity of growing GIST lesions [13]. In GIST of the stomach, adopting the standard therapy of epithelial gastric cancer, i.e., total gastrectomy may produce a conflict with adjuvant therapy as imatinib plasma levels are significantly below the therapeutic threshold [14].

The use of IM in the neoadjuvant setting can play an important role by downsizing the tumor, in this way decreasing the extent of resection (i.e., organ-preserving operation) [15,16,17,18,19,20,21,22,23,24,25]. Particularly in cases of gastric GISTs, neoadjuvant IM therapy may also convert surgical procedures from an open to a laparoscopic approach.

The purpose of this study was to evaluate the clinic-pathological profile and the surgical and oncological outcomes of patients with gastric GIST who underwent a neoadjuvant IM therapy followed by surgery from a prospectively kept database. We particularly were interested in analyzing a subgroup of patients who had started neoadjuvant therapy in the clinical setting of acute upper GI bleeding from imatinib-sensitive gastric GIST.

## 2. Material and Methods

### 2.1. Patient Selection

**Figure 1:**
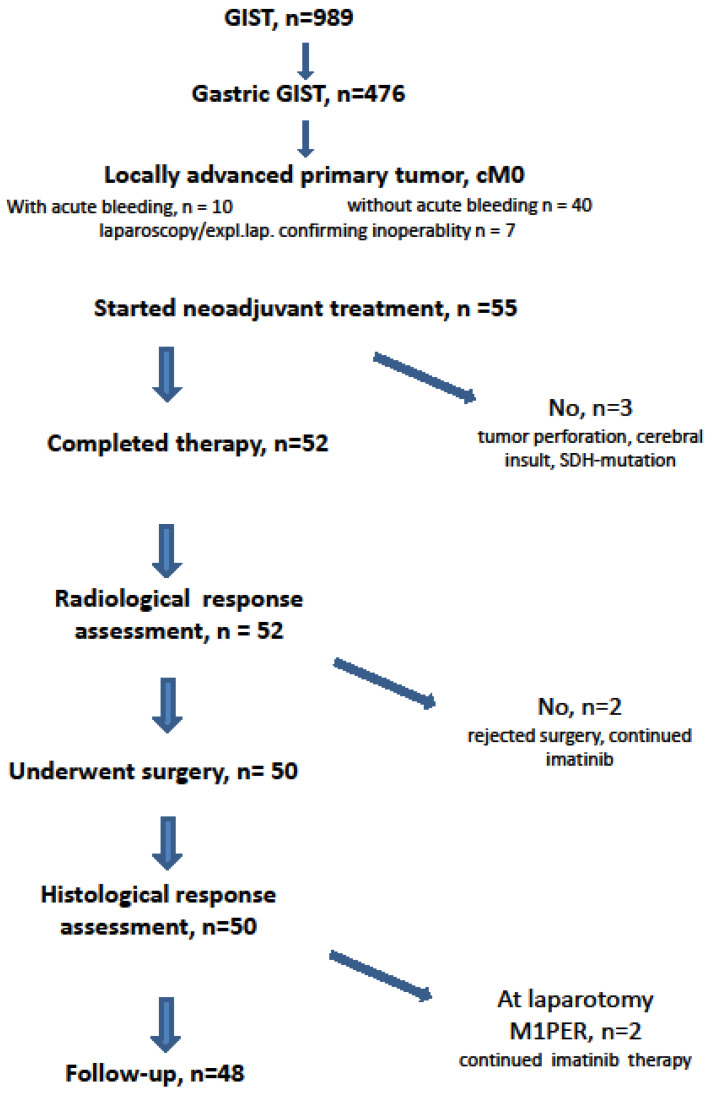
CONSORT statement.

From November 2002 to December 2019, 989 patients with histologically proven GIST were treated by one therapeutic surgical team (PH). Of these, 476 patients had the primary GIST originating from the stomach(Figure 1). In addition to a prospective phase II neoadjuvant study (NCT00112632) [23], we subjected patients who had locally advanced, histopathologically proven gastric GISTs to the similar protocol. The indication was given when patients would have required extensive surgeries (total gastrectomy or multivisceral resection) for curative treatment or when the tumors were ill-located (e.g., GIST at the esophagogastric junction) requiring an abdomino-thoracic approach [26]. Patients with metastatic disease at time of diagnosis or patients treated because of local recurrence of gastric GIST were not included in this analysis.

### 2.2. Clinical Condition

**Figure 2:**
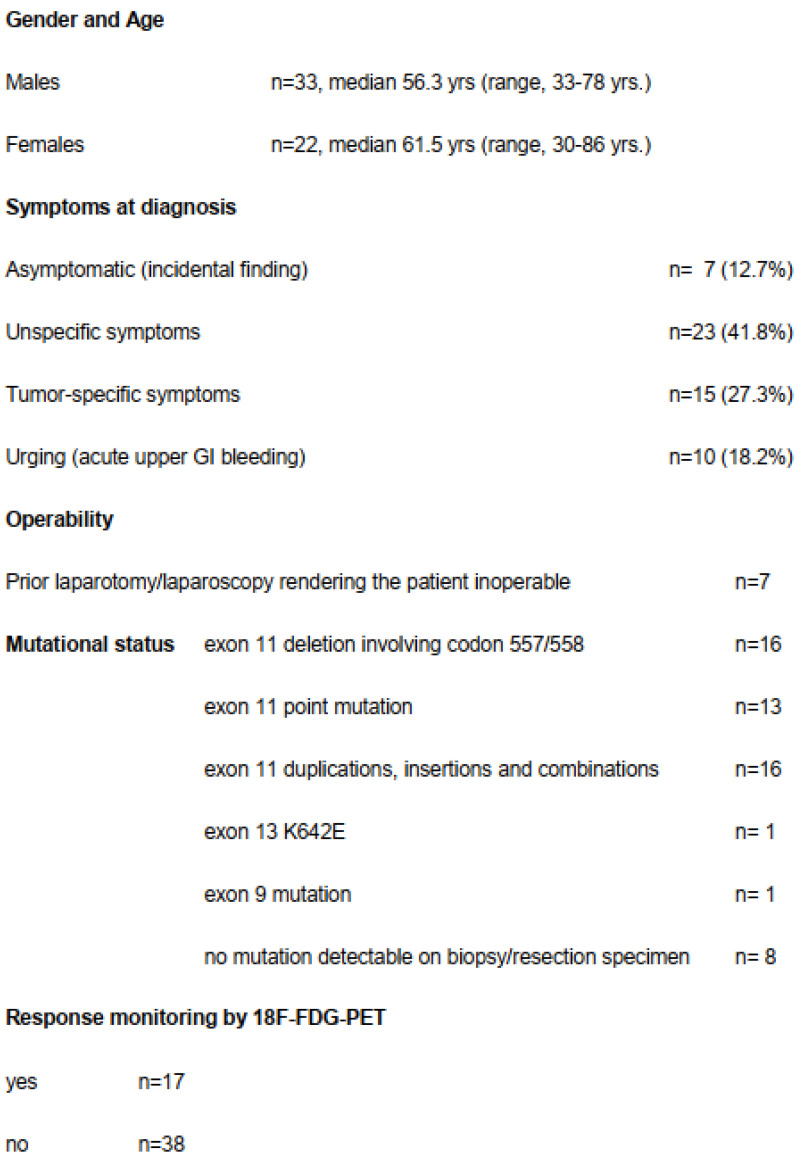
Demographic, clinical and histopathological data.

Only seven patients (12.7%) were asymptomatic and the tumor was detected incidentally (Figure 2). Seven patients had already undergone an exploration (exploratory laparotomy (*n* = 3) or diagnostic laparoscopy (*n* = 4)) at another hospital declaring inoperability or tumor resection with only multivisceral procedure and therefore had been referred to our institution.

It is of note that in 10 patients (18.2%), the GIST was diagnosed due to an acute upper GI bleeding. When there was suspicion on endoscopy and abdominal CT scan, we immediately started with imatinib after endoscopical control of the bleeding and tumor biopsy. Patients who suffered from subacute melena or occult fecal bleeding prompting the diagnosis of GIST were subsumed in the subgroup of tumor-specific symptoms.

### 2.3. Imatinib Mesylate Therapy and Response Assessment

The treatment plan of each patient was managed by a multidisciplinary GIST team consisting of surgical oncologists, medical oncologists, pathologists and radiologists. Before treatment started, tumor biopsy was obtained and all patients had confirmed diagnosis of GIST. Mutation analysis was always carried-out when enough tissue material was available. Risk stratification into very low, low, intermediate and high risk followed the NIH-Fletcher criteria for GIST risk assessment [1].

Imatinib was given orally at 400 mg per day, as a single daily dosing; one patient with KIT exon 9 mutation received 800 mg of imatinib. The duration of IM therapy was intended to last 6 months or as long as the tumor was still shrinking in size.

Response to neoadjuvant IM therapy was evaluated 1 month after the treatment start and then every 3 months either with positron emission computed tomography (PET-CT), dual-energy computed tomography (DE-CT) or contrast-enhanced magnetic resonance imaging (CE-MRI). PET imaging was only used to make sure that the tumor would respond to imatinib. Thus, it was of importance in the earlier patients when the results of mutational analysis took longer than it does today. Response was determined according to the Response Evaluation Criteria In Solid Tumors version 1.0 and version 1.1 (RECIST 1.1) as a complete response (CR), partial response (PR), stable disease (SD) or progressive disease (PD) [27].

### 2.4. Conduct of Surgery

Based on the imaging data, removal of the residual tumor was indicated when the maximum therapeutic response was reached (no further reduction in tumor diameters in consecutive imagings of 3 months) or when no further influence on the resectional strategy was expected. A margin of safety of 1 cm was considered enough to spare organ function [28]. The type of surgery was classified according to EORTC STBSG classification: local excision (wedge resection), limited resection (partial resection of the stomach), typical organ resection (total gastrectomy) and multivisceral resection (including adjacent organs) or other (with verbal specification) [29]. Postoperative complications were classified using the Clavien–Dindo classification [30].

The extent of tumor regression was measured at the resection specimen. Complete remission was defined as 100% necrosis (complete absence of viable tumor cells), a near total remission was defined as 95–99% necrosis, subtotal 90–95%, partial remission with 50–90% necrosis and stable disease with <50% necrosis. Resection margin status was defined as R0, R1 and R2 [31].

### 2.5. Postoperative Drug Therapy

There was no stringent policy regarding drug treatment after residual tumor resection. We did not continue imatinib in patients with >95% regression of the tumor and discussed further drug therapy on an individual basis.

### 2.6. Follow-Up

Postoperative follow-up consisted of a physical examination and acquisition of DE-CT at 3-month intervals for the first 2 years, every 6 months for the next 3 years and yearly thereafter for the next 5 years. Recurrence was defined as recurrent disease in the region of the previously located tumor. Metastasis was defined as disease in distant sites, predominantly liver and peritoneum. All patients were followed-up for a median of 82 months (range, 3–182 months) and were last updated in June 2020.

### 2.7. Statistical Analysis

The statistical analysis of the prospectively maintained database was performed with SPSS (version 21). Survival outcomes in terms of RFS was analyzed. RFS was calculated from the date of surgical resection to the date of clinical or radiological evidence of disease relapse, last follow-up or death, whichever occurred first. RFS percentages and treatment effect comparisons were obtained from the Kaplan–Meier method [32] and log-rank test [33]. Date is given as median with range or mean +/− standard deviation. Correlative analytics were obtained by Pearson and Spearman rank-coefficient tests. Differences were considered statistically significant when *p* ≤ 0.05.

## 3. Results

### 3.1. Demographic and Clinicopathological Data

Fifty-five patients (22f, 33m) with a median age of 58.2 years, (range, 30–86 years) were included in this study (Table 1). Detailed demographic, clinical and histopathological data are listed in Table 1. The median tumor size before start of imatinib was 113 mm (range, 65–330 mm). Mitotic index could be determined in 41 patients (75%); in the remaining patients the size of the biopsy did not allow us to count enough high-power fields (HPFs). According to National Institutes of Health (NIH) consensus [1], the risk classification was “high risk” in 26 patients (48%), “intermediate risk” in 15 patients (28%) and “low risk” in 13 patients (24%).

### 3.2. Mutational Data

Mutation analysis was performed in 49 patients and showed exon 11 mutation in 47 patients with the majority of the mutations consisting of deletions involving codons 557 and 558 (*n* = 15) or point mutation (*n* = 12) (Table 1). In one patient, a SDHB mutation was determined after 2 months of imatinib and the patient was operated on immediately. In six patients mutation analysis could not be carried out from the biopsy and even at the resection specimen it was not feasible due to complete tumor necrosis and significant tumor shrinkage. In another patient who had significant tumor shrinkage, a K642E mutation at KIT exon 13 was found with NGS-sequencing from the residual tumor mass.

### 3.3. Imatinib Therapy and Clinical Response

The median time of preoperative imatinib therapy was 10 months (range, 2–21 months) and 52 patients (94.5%) completed the expected treatment duration. Among the patients who did not complete therapy there was the patient with the SDHB mutation whose treatment had to be stopped and a male patient of 69 years (with a history of vascular occlusive disease) who died from a cerebral insult 3 weeks after start of imatinib. Another patient developed perforation of the tumor, located at the posterior part of the stomach, 8 weeks after start of treatment due to extensive tumor regression. He underwent emergency subtotal gastrectomy, partial resection of the diaphragm and splenectomy. Another patient experienced grade 3 skin toxicity which could be resolved by switching therapy to nilotinib. Grade 2 side effects included skin toxicity (*n* = 1) and depression (*n* = 2).

The median tumor size prior to surgery shrank to 62 mm (range, 22–200 mm). According to RECIST 1.1, 39 patients had a PR (75%) while 14 patients had SD (25%) and no patient had progressive disease, see Table 1.

All 10 patients who started neoadjuvant therapy with acute upper GI bleeding experienced no further episode of bleeding and completed their drug schedule until surgery.

### 3.4. Surgical Data

Fifty patients underwent surgery after achieving the plateau response. Except for the patient who experienced tumor perforation, all others were operated on at the intended date. Two patients refused surgery. Of these two, one patient preferred to continue with imatinib while another one committed suicide.

**Figure 3:**
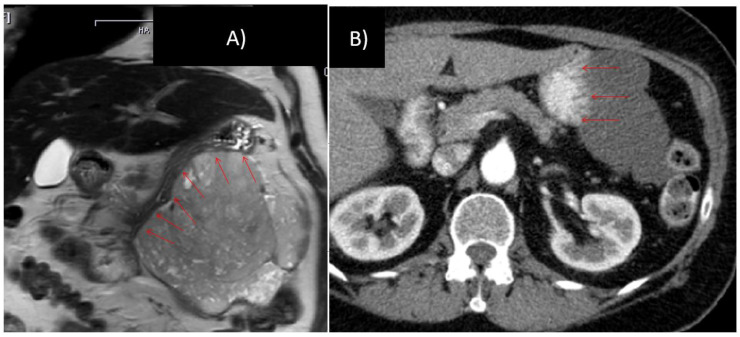
76 year old female, gastrointestinal stromal tumors (GIST) with broad contact to the greater curvature, scheduled for total gastrectomy, left hemicolectomy and left-sided pancreatico-/splenectomy (**A**) prior to and (**B**) after 10 months of imatinib therapy.

**Figure 4:**
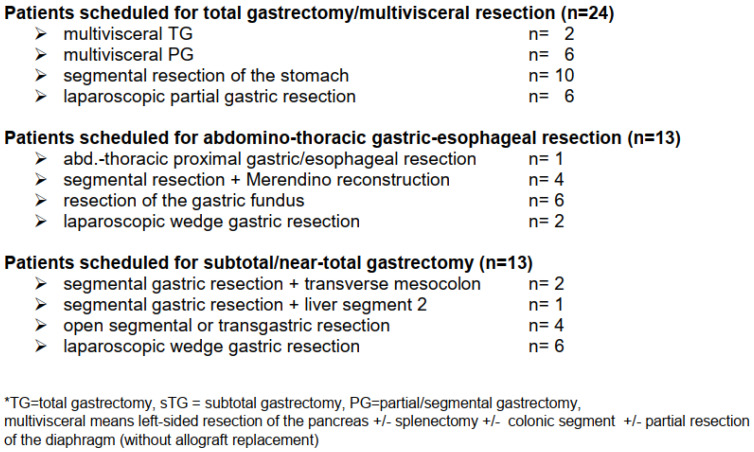
Comparison of scheduled surgery prior to imatinib vs. surgical procedure performed after neoadjuvant therapy.

At laparotomy, in two patients previously undetected peritoneal metastases were found at the bursa omentalis and the omentum. In 48 patients, total gastrectomy or abdomino-thoracic resection could be avoided, resulting in a stomach preservation rate of 96% (see Figure 3 and Figure 4). The details of the surgical procedures are illustrated in Figure 4 (e.g., treatment option before IM vs. surgical procedure after IM). In 12 cases (24%), the downstaging of the tumor allowed a laparoscopic procedure instead of open laparotomy.

All patients having undergone prior exploratory laparotomy and declared inoperable (*n* = 3) could be resected with clear margins by multivisceral (MVR) total gastrectomy, total gastrectomy alone and subtotal gastrectomy in one patient each. In the four patients who had prior diagnostic laparoscopy (MVR only), segmental resection with Merendino reconstruction (*n* = 1), subtotal (*n* = 1) or segmental (*n* = 1) gastric resection, and MVR (*n* = 1) had to carried out for R0 tumor removal.

Surgical complications were observed in seven patients (14%) and included postoperative pancreatic fistula (*n* = 2), surgical site hemorrhage (*n* = 2), prolonged pleural effusion (*n* = 2) and wound infection (*n* = 1). Of them, three patients required intervention (grade 3 Clavien–Dindo). None of these patients required reoperation and there was no postoperative mortality.

### 3.5. Histopathological Data

Except for the two patients with peritoneal metastases (R2 resection), only in one patient was an R1 resection stated by the pathologist, resulting in an R0 resection rate of 47/50 patients (94%).

The final histopathological report showed no residual viable tumor (pCR) in 12 cases (24%); for details see Table 1. Interestingly, in seven cases there was a less than 50% necrosis observed, despite the fact that even in this subgroup the median tumor size shrank from 84 mm (range, 65–122 mm) to 60 mm (range, 22–115 mm).

Only the mutational type correlates significantly (*p* = 0.037) with the extent of tumor regression. There was no significant relationship of RECIST classification to the difference in tumor size from prior to imatinib vs. post-imatinib (Table 1). The difference between tumor size prior to imatinib vs. at the resection specimen exceeded *p* = 0.05.

### 3.6. Recurrence-Free Survival

Of the 50 patients operated, 34 (68%) are alive with no evidence of disease. The most common sites at detection of distant metastases were peritoneum (*n* = 7) and liver (*n* = 6), both combined (*n* = 2), while one patient developed a locoregional recurrence at the surgical site. Three patients have died from their disease, and another four patients from other causes.

**Figure 5:**
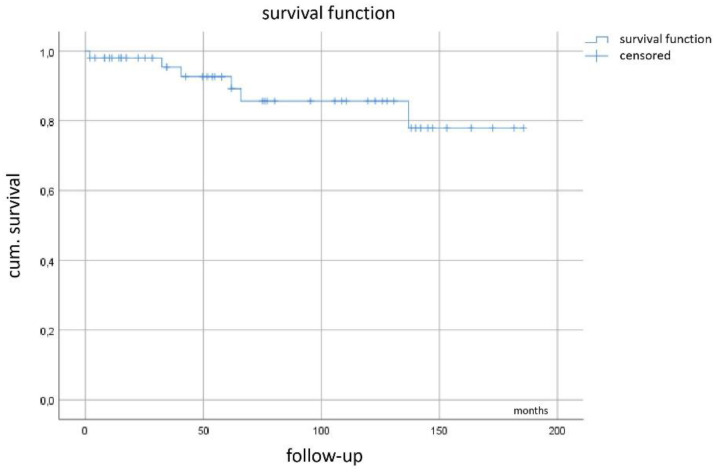
Recurrence-free survival.

Kaplan–Meier curve (Figure 5) demonstrates a mean recurrence-free survival of 123 months (95%CI 99–147 months) with the median not yet reached.

We evaluated prognostic factors with respect to the influence on RFS. However, no statistically significant result could be obtained from initial tumor size (*p* = 0.34), mutational type (*p* = 0.86) and mitotic count (*p* = 0.12). Furthermore, the most logical factor (extent of tumor necrosis) was not proven to be of significant influence (*p* = 0.33, all log-rank).

With respect to adjuvant imatinib therapy after residual tumor removal, the single patient who experienced tumor perforation continued with imatinib and is free from recurrence after 12 years. Another seven patients were recommended to continue with imatinib therapy in order to complete a 36 months total duration of neoadjuvant plus adjuvant drug therapy. Of these, three patients developed hepatic and/or peritoneal metastases and died from their disease after multiple lines of therapy. Another two patients completed 3 years with no evidence of recurrence, one patient chose to stick with the drug until now and one patient is still in the completion phase.

## 4. Discussion

Surgery for primary GIST of the stomach is different from surgery for epithelial gastric cancer. Detailed lymphadenectomy, the mainstay to treat gastric carcinoma, is not required except in a subgroup of patients with Carney–Stratakis syndrome or SDH-deficient tumors presenting typically in young females [34]. On the other hand, GISTs originating from the muscularis layer of the intestine tend to grow luminally with potential acute bleeding or exophytically towards the surrounding organs. As GIST may be a fragile mass and often represents a highly vascularized lesion, larger gastric GIST may require more extended surgery with major morbidity and functional deficits due to the proximity to vital structures or the location in difficult sites (e.g., gastroesophageal junction). This may be the indication for imatinib therapy prior to surgery as recommended by several guidelines [35]. The neoadjuvant administration of IM turns out to be beneficial for patients with locally advanced or marginally resectable, non-metastatic GIST. Proper selection of candidates for neoadjuvant therapy is the prerequisite for successful therapy and requires tumor genotyping based on preoperative biopsy with the mutational spectrum not different from the metastatic situation [36]. In our study, all patients were either diagnosed with imatinib-sensitive mutations of *KIT* or we used ^18^F-FDG-PET to make sure the expected efficacy would really take place. This was particularly the case in the earlier patients when mutational analysis took more time than today [37,38].

The few formal trials on preoperative imatinib therapy often include both locally advanced and metastatic patients with GIST arising from the whole GI tract [15,21,24,36,39,40,41,42]. In the RTOG study [24] only 15 patients were truly treated under neoadjuvant conditions across all locations. Thus, our series comprises the largest patient cohort of locally advanced, non-metastatic gastric GIST patients treated with neoadjuvant IM therapy followed by surgery. Large GISTs carry an increased risk of intraoperative tumor rupture and dissemination because of their fragility and hypervascularity which has a detrimental effect on disease-free status and overall survival [43,44,45,46]. Beyond the organ-saving approach through objective tumor downsizing, preoperative imatinib also improves the integrity of tumor capsule and decreases the risk of intraperitoneal bleeding/tumor perforation, leading to a very high rate of R0 resections [21,46]. We demonstrate a significant regression of median tumor size from 11.3 cm to 6.2 cm, which reflects the main advantage of imatinib as induction therapy in patients with locally advanced GIST. Particularly the subgroup of 10 patients (18.2%) with upper GI bleeding from the tumor profited from this approach. None of them had to be operated on prematurely due to a recurrent bleeding episode and the surgical tumor resection could be moved from an emergency procedure to an elective operation. No postoperative imatinib-related complications were observed.

The approach provided an excellent oncological long-term result. Based on the NIH consensus criteria [1], 48% of the patients could be classified as high risk for tumor recurrence. Even if one uses the contour maps by Joensuu et al. [47], providing a better assessment tool and eliminating the dichotomous threshold of 5 cm and 5 mitoses pro 50 HPF, the mean recurrence-free survival of more than 10 years looks very promising. The basis probably is laid by the fact that 44% of the resection specimen showed >95% necrosis and 94% of the patients have undergone R0 resection. This is due to patient selection with *KIT* exon 11 mutations almost exclusively. It is also known from treating metastatic patients that KIT deletions involving codons 557 and 558 respond very well to imatinib [48] (Figure 2). Our data are in line with a multicenter study including 161 patients with locally advanced non-metastatic GISTs pooled from 10 EORTC-STBSG sarcoma centers showing that >80% of the tumors responded to imatinib, facilitating R0 resection in >80% of the cases [24]. After a median 40 weeks of imatinib, the R0 resection rate was 83% and the 5-year DFS was 65% with median OS of 104 months [24].

Another recently published series on 150 patients with GIST treated on a neoadjuvant basis across all tumor sites reports an overall survival rate of 81% at 5 years [49]. The difference might be due to shorter treatment duration (median 7.1 months with a range starting at 0.2 months) and a clearly lower rate of partial tumor remissions of 40% which was 75% in our series. Furthermore, the resection margins with 63.3% R0 resections and 18% each of R1 and R2 resection are inferior to our study [49]. It has been noted from further studies that patients after R0 resection have a significantly lower risk of developing tumor progression compared to patients with R1/R2 resection (60% vs. 23.8%, *p* = 0.11, [22,40]).

The duration of neoadjuvant imatinib administration may be important to obtain adequate tumor response. An early compilation of case reports by Haller et al. [50] suggested that the longer the treatment the better the remission. We indicated surgery after having reached a plateau with no further tumor shrinkage and the risk of developing secondary resistance to therapy still remaing low [39]. At this time point, all our patients showed either PR or SD and no patient showed any progression during imatinib therapy. The rate of partial responses in our patients is higher compared with the phase II RTOG 0132 trial, in which 83% of patients had stable disease after 12 months of imatinib [15].

A strength of this study is that it demonstrates that laparoscopic procedures more and more can be successfully used in this setting of locally advanced GIST with median tumor size of more than 10 cm after downstaging with tyrosine kinase inhibitors. The study, however, also has limitations referring mainly to patient selection which is hardly avoidable. Patients can easily be convinced to swallow a pill per day and avoid total gastrectomy or multivisceral resection. A randomized trial does not look feasible at all under these circumstances and therefore the formal evidence of using neoadjuvant imatinib is not better than grade 2+ according to SIGN^+1^ [51]. 

Given the fact that in small tumor biopsies the number of mitoses could not be counted per 5 mm^2^ or 50 HPF, the risk classification of patients according to NIH or Miettinen/Lasota is doubtful in some cases. This also influences the decision of whether or not to subject patients to postoperative adjuvant imatinib therapy. Using the extent of regression from the resection specimen in the seven mentioned patients does not allow us to draw conclusions. The willingness of the patients to continue with the drug also influenced the administration of adjuvant imatinib therapy. Several patients felt relief from the drug and the tumor after surgery and were not willing to continue. Our individualized approach does not allow us to draw further conclusions.

In gastric GIST a problem is the rather high rate of tumors without mutations in KIT or with mutations in PDGFRA. We tried to overcome this with ^18^F-FDG-PET scanning to eliminate patients who would not respond adequately. We also postponed patients with epitheloid GIST until mutation analysis had been performed, as this feature is often associated with PDGFRA mutations not being sensitive to imatinib.

## 5. Conclusions

In conclusion, neoadjuvant imatinib in our series of locally advanced gastric GIST proved to allow organ-sparing surgical procedures with a very high rate of R0 resections and excellent long-term recurrence-free survival. This holds true also for patients starting their treatment during an episode of upper gastrointestinal bleeding. Toxicity was mild and tolerable and in 96% of the patients major parts of the stomach could be preserved, maintaining the physiological basis for the use of oral tyrosine kinase inhibitors in the adjuvant or metastatic setting.

## Figures and Tables

**Table 1:** Analysis of Response Data.

**Tumor Size**			
Tumor size at start of treatment: Tumor size prior to surgery:	113 mm (range, 65–330 mm) (measured by CT/MRI) 69 mm (range, 25–228 mm) (measured by CT/MRI)		
Tumor size at resection specimen:	62 mm (range, 22–200 mm) (measured by pathology)		
**Pathology Review of the Resection Specimen**			
Complete necrosis (no viable tumor cells)	*n* = 12 (24%)		
Near total (>95% necrosis)	*n* = 10 (20%)		
Subtotal (>90% necrosis)	*n* = 7 (14%)		
Partial remission (>50% necrosis)	*n* = 14 (28%)		
Stable disease (<50% necrosis)	*n* = 7 (14%)		
**Correlation of Response to Therapy** **(Pearson, Two-Sided; Spearman)**			
Δtumor diameter pre vs. post		*p* = 0.078	*p* = 0.089
RECIST 1.1		*p* = 0.2	*p* = 0.21
Mutational type exon 11		*p* = 0.04	*p* = 0.037
	point mutation		
	del involving codons 557_558		
	others		

## Data Availability

The data that support the findings of this study are available from the corresponding author upon reasonable request.

## References

[1] Fletcher C.D., Berman J.J., Corless C., Gorstein F., Lasota J., Longley B.J., Miettinen M., O’Leary T.J., Remotti H., Rubin B.P. (2002). Diagnosis of gastrointestinal stromal tumors: A consensus approach. Hum. Pathol..

[2] Nilsson B., Bümming P., Meis-Kindblom J.M., Oden A., Dortok A., Gustavsson B., Sablinska K., Kindblom L.G. (2005). Gastrointestinal stromal tumors: The incidence, prevalence, clinical course, and prognostication in the preimatinib mesylate era—A population-based study in western Sweden. Cancer.

[3] DeMatteo R.P., Lewis J.J., Leung D., Mudan S.S., Woodruff J.M., Brennan M.F. (2000). Two hundred gastrointestinal stromal tumors: Recurrence patterns and prognostic factors for survival. Ann. Surg..

[4] Miettinen M., Lasota J. (2006). Gastrointestinal stromal tumors: Review on morphology, molecular pathology, prognosis, and differential diagnosis. Arch. Pathol. Lab. Med..

[5] Hirota S. (1998). Gain-of-function Mutations of c-kit in human gastrointestinal stromal tumors. Science.

[6] Heinrich M.C., Corless C.L., Duensing A., McGreevey L., Chen C.J., Joseph N., Singer S., Griffith D.J., Haley A., Town A. (2003). PDGFRA activating mutations in gastrointestinal stromal tumors. Science.

[7] Roberts P.J., Eisenberg B. (2002). Clinical presentation of gastrointestinal stromal tumors and treatment of operable disease. Eur. J. Cancer.

[8] Dematteo R.P., Heinrich M.C., El-Rifai W.M., Demetri G. (2002). Clinical management of gastrointestinal stromal tumors: Before and after STI-571. Hum. Pathol..

[9] Joensuu H., Roberts P.J., Sarlomo-Rikala M., Andersson L.C., Tervahartiala P., Tuveson D., Silberman S., Capdeville R., Dimitrijevic S., Druker B. (2001). Effect of the tyrosine kinase inhibitor STI571 in a patient with a metastatic gastrointestinal stromal tumor. N. Engl. J. Med..

[10] Heinrich M.C., Blanke C.D., Druker B.J., Corless C.L. (2002). Inhibition of KIT Tyrosine Kinase Activity: A Novel Molecular Approach to the Treatment of KIT-Positive Malignancies. J. Clin. Oncol..

[11] Demetri G.D., Demetri G.D., von Mehren M., Blanke C.D., Van den Abbeele A.D., Eisenberg B., Roberts P.J., Heinrich M.C., Tuveson D.A., Singer S. (2002). Efficacy and safety of imatinib mesylate in advanced gastrointestinal stromal tumors. N. Engl. J. Med..

[12] van Oosterom A.T., Judson I., Verweij J., Stroobants S., Donato di Paola E., Dimitrijevic S., Martens M., Webb A., Sciot R., van Glabbeke M. (2001). Safety and efficacy of imatinib (STI571) in metastatic gastrointestinal stromal tumours: A phase I study. Lancet.

[13] Demetri G.D., von Mehren M., Antonescu C.R., DeMatteo R.P., Ganjoo K.N., Maki R.G., Pisters P.W.T., Raut C.P., Riedel R.F., Schuetze S. (2010). NCCN Task Force report: Update on the management of patients with gastrointestinal stromal tumors. J. Natl. Compr. Cancer Netw..

[14] Demetri G.D., Wang Y., Wehrle E., Racine A., Nikolova Z., Blanke C.D., Joensuu H., von Mehren M. (2009). Imatinib plasma levels are correlated with clinical benefit in patients with unresectable/metastatic gastrointestinal stromal tumors. J. Clin. Oncol..

[15] Eisenberg B.L., Harris J., Blanke C.D., Demetri G.D., Heinrich M.C., Watson J.C., Hoffman J.P., Okuno S., Kane J.M., von Mehren M. (2009). Phase II trial of neoadjuvant/adjuvant imatinib mesylate (IM) for advanced primary and metastatic/recurrent operable gastrointestinal stromal tumor (GIST): Early results of RTOG 0132/ACRIN 6665. J. Surg. Oncol..

[16] Shrikhande S.V., Marda S.S., Suradkar K., Arya S., Shetty G.S., Bal M., Shukla P.J., Goel M., Mohandas K.M. (2012). Gastrointestinal stromal tumors: Case series of 29 patients defining the role of imatinib prior to surgery. World J. Surg..

[17] Koontz M.Z., Visser B.M., Kunz P.L. (2012). Neoadjuvant imatinib for borderline resectable GIST. J. Natl. Compr. Cancer Netw..

[18] Tielen R., Verhoef C., van Coevorden F., Gelderblom H., Sleijfer S., Hartgrink H.H., Bonenkamp J.J., van der Graaf W.T., de Wilt J.H. (2013). Surgical treatment of locally advanced, non-metastatic, gastrointestinal stromal tumours after treatment with imatinib. Eur. J. Surg. Oncol..

[19] Fiore M., Palassini E., Fumagalli E., Pilotti S., Tamborini E., Stacchiotti S., Pennacchioli E., Casali P.G., Gronchi A. (2009). Preoperative imatinib mesylate for unresectable or locally advanced primary gastrointestinal stromal tumors (GIST). Eur. J. Surg. Oncol..

[20] Andtbacka R.H., Ng C.S., Scaife C.L., Cormier J.N., Hunt K.K., Pisters P.W.T., Pollock R.E., Benjamin R.S., Burgess M.A., Chen L.L. (2007). Surgical resection of gastrointestinal stromal tumors after treatment with imatinib. Ann. Surg. Oncol..

[21] Raut C.P., Posner M., Desai J., Morgan J.A., George S., Zahrieh D., Fletcher C.D.M., Demetri G.D., Bertagnolli M.M. (2006). Surgical management of advanced gastrointestinal stromal tumors after treatment with targeted systemic therapy using kinase inhibitors. J. Clin. Oncol..

[22] Blesius A., Cassier P.A., Bertucci F., Fayette J., Ray-Coquard I., Bui B., Adenis A., Rios M., Didier C., Perol D. (2011). Neoadjuvant imatinib in patients with locally advanced non metastatic GIST in the prospective BFR14 trial. BMC Cancer.

[23] Hohenberger P., Langer C., Wendtner C.M., Hohenberger W., Pustowka A., Wardelmann E., Andre E., Licht T. (2012). Neoadjuvant treatment of locally advanced GIST: Results of APOLLON, a prospective, open label phase II study in KIT- or PDGFRA-positive tumors. J. Clin. Oncol..

[24] Rutkowski P., Gronchi A., Hohenberger P., Bonvalot S., Schoffski P., Bauer S., Fumagalli E., Nyckowski P., Nguyen B.P., Kerst J.M. (2013). Neoadjuvant Imatinib in Locally Advanced Gastrointestinal Stromal Tumors (GIST): The EORTC STBSG Experience. Ann. Surg. Oncol..

[25] Wang S.Y., Wu C.E., Lai C.C., Chen J.S., Tsai C.Y., Cheng C.T., Yeh T.S., Yeh C.N. (2019). Prospective evaluation of preoperative IM use in locally advanced gastrointestinal stromal tumors: Emphasis on the optimal duration of preoperative IM use, safety, and oncological outcome. Cancers.

[26] Staiger W.I., Ronellenfitsch U., Kaehler G., Schildhaus H.U., Dimitrakopoulou-Strauss A., Schwarzbach M.H., Hohenberger P. (2008). The Merendino procedure following preoperative imatinib mesylate for locally advanced gastrointestinal stromal tumor of the esophagogastric junction. World J. Surg. Oncol..

[27] Eisenhauer E.A., Therasse P., Bogaerts J., Schwartz L.H., Sargent D., Ford R., Dancey J., Arbuck S., Gwyther S., Mooney M. (2009). New response evaluation criteria in solid tumours: Revised RECIST guideline (version 1.1). Eur. J. Cancer.

[28] Rutkowski P., Skoczylas J., Wisniewski P. (2018). Is the surgical margin in gastrointestinal stromal tumors different?. Visc. Med..

[29] Hohenberger P., Bonvalot S., van Coevorden F., Rutkowski P., Stoeckle E., Olungu C., Litiere S., Wardelmann E., Gronchi A., Casali P. (2019). Quality of surgery and surgical reporting for patients with primary gastrointestinal stromal tumors participating in the EORTC STBSG 62024 adjuvant imatinib study. Eur. J. Cancer.

[30] Dindo D., Demartines N., Clavien P.A. (2004). Classification of Surgical Complications: A New Proposal With Evaluation in a Cohort of 6336 Patients and Results of a Survey. Ann. Surg..

[31] Hermanek P., Wittekind C. (1994). The pathologist and the residual tumor (R) classification. Pathol. Res. Pract..

[32] Kaplan E.L., Meier P. (1958). Nonparametric estimation from incomplete observations. J. Am. Stat. Assoc..

[33] Mantel N. (1966). Evaluation of survival data and two new rank order statistics arising in its consideration. Cancer Chemother. Rep..

[34] Bachet J.B., Landi B., Laurent-Puig P., Italiano A., Le Cesne A., Levy P., Safar V., Duffaud F., Blay J.Y., Emile J.F. (2013). Diagnosis, prognosis and treatment of patients with gastrointestinal stromal tumour (GIST) and germline mutation of KIT exon 13. Eur. J. Cancer.

[35] Casali P.G., Abecassis N., Aro H.T., Bauer S., Biagini R., Bielack S., Boukovinas I., Bovee J.V.M.G., Brodowicz T., Brotto J.M. (2018). Gastrointetinal stromal tumors: ESMO-EURACAN clinical practice guidelines for diagnosis, treatment and follow-up. Ann. Oncol..

[36] Jakob J., Hohenberger P. (2018). Neoadjuvant therapy to downstage the extent of resection of gastrointestinal stromal tumors. Visc. Med..

[37] Choi H. (2008). Response evaluation of gastrointestinal stromal tumors. Oncologist.

[38] Van den Abbeele A.D. (2008). The lessons of GIST–PET and PET/CT: A new paradigm for imaging. Oncologist.

[39] Bonvalot S., Eldweny H., Pechoux C.L., Vanel D., Terrier P., Cavalcanti A., Robert C., Lassau N., Cesne A.L. (2006). Impact of Surgery on Advanced Gastrointestinal Stromal Tumors (GIST) in the Imatinib Era. Ann. Surg. Oncol..

[40] Wang D., Zhang Q., Blanke C.D., Demetri G.D., Heinrich M.C., Watson J.C., Hoffman J.P., Okuno S., Kane J.M., von Mehren M. (2011). Phase II Trial of Neoadjuvant/adjuvant Imatinib Mesylate for Advanced Primary and Metastatic/recurrent Operable Gastrointestinal Stromal Tumors: Long-term Follow-up Results of Radiation Therapy Oncology Group 0132. Ann. Surg. Oncol..

[41] Gronchi A., Fiore M., Miselli F., Lagonigro M.S., Coco P., Messina A., Pilotti S., Casali P.G. (2007). Surgery of residual disease following molecular-targeted therapy with imatinib mesylate in advanced/metastatic GIST. Ann. Surg..

[42] Rutkowski P., Nowecki Z., Nyckowski P., Dziewirski W., Grzesiakowska U., Nasierowska-Guttmejer A., Krawczyk M., Ruka W. (2006). Surgical treatment of patients with initially inoperable and/or metastatic gastrointestinal stromal tumors (GIST) during therapy with imatinib mesylate. J. Surg. Oncol..

[43] Hohenberger P., Ronellenfitsch U., Oladeji O., Pink D., Ströbel P., Wardelmann E., Reichardt P. (2010). Pattern of recurrence in patients with ruptured primary gastrointestinal stromal tumour. Br. J. Surg..

[44] Gronchi A., Raut C.P. (2012). The combination of surgery and imatinib in GIST: A reality for localized tumors at high risk, an open issue for metastatic ones. Ann. Surg. Oncol..

[45] Hohenberger P., Eisenberg B. (2010). Role of surgery combined with kinase inhibition in the management of gastrointestinal stromal tumor (GIST). Ann. Surg. Oncol..

[46] Tang S., Yin Y., Shen C., Chen J., Yin X., Zhang B., Yao Y., Yang J., Chen Z. (2017). Preoperative imatinib mesylate (IM) for huge gastrointestinal stromal tumors (GIST). World J. Surg. Oncol..

[47] Joensuu H., Vehtari A., Riihimäki J., Nishida T., Steigen S.E., Brabec P., Plank L., Nilsson B., Cirilli C., Braconi C. (2012). Risk of recurrence of gastrointestinal stromal tumour after surgery: An analysis of pooled population-based cohorts. Lancet Oncol..

[48] McAuliffe J.C., Hunt K.K., Lazar A.J., Choi H., Qiao W., Thall P., Pollock R.E., Benjamin R.S., Trent J.C. (2009). A randomized, phase II study of preoperative plus postoperative imatinib in GIST: Evidence of rapid radiographic response and temporal induction of tumor cell apoptosis. Ann. Surg. Oncol..

[49] Cavnar M.J., Seier K., Gönen M., Curtini C., Balachandran V.P., Tap W.D., Antonescu C.R., Singer S., DeMatteo R.P. (2020). Prognostic factors after neoadjuvant imatinib for newly diagnosed primary gastrointestinal stromal tumor. J. Gastrointest. Surg..

[50] Haller F., Detken S., Schulten H.J., Happel N., Gunawan B., Kuhlgatz J., Füzesi L. (2007). Surgical management after neoadjuvant imatinib therapy in gastrointestinal stromal tumours (GISTs) with respect to imatinib resistance caused by secondary KIT mutations. Ann. Surg. Oncol..

[51] Edinburgh: Scottis Intercollegiate Guidelines Network. http://www.sign.ac.uk/.

